# 1,1′-Dimethyl-4,4′-(2,4-di-1-naphthyl­cyclo­butane-1,3-di­yl)dipyridinium–(*E*)-1-methyl-4-[2-(1-naphth­yl)vin­yl]pyridinium–4-amino­benzene­sulfonate–water (0.25/1.50/2/2)

**DOI:** 10.1107/S1600536809029730

**Published:** 2009-07-31

**Authors:** Hoong-Kun Fun, Kullapa Chanawanno, Suchada Chantrapromma

**Affiliations:** a X-ray Crystallography Unit, School of Physics, Universiti Sains Malaysia, 11800 USM, Penang, Malaysia; b Crystal Materials Research Unit, Department of Chemistry, Faculty of Science, Prince of Songkla University, Hat-Yai, Songkhla 90112, Thailand

## Abstract

In the title compound, 1.5C_18_H_16_N^+^·0.25C_36_H_32_N_2_^2+^·2C_6_H_6_NO_3_S^−^·2H_2_O, the monocation exists in the *E* configuration with respect to the ethenyl C=C double bond and is almost planar, the dihedral angles between the pyridinium and the fused six-membered rings being 3.1 (7) and 3.8 (8)°. The dication lies about an inversion centre. In the crystal, the dication occupies almost the same site occupied by monocations at (*x*, *y*, *z*) and (−*x*, 1 − *y*, 1 − *z*). The anions and water mol­ecules are linked into a chain along the *a* axis by O—H⋯O and N—H⋯O hydrogen bonds. The structure is further stabilized by C—H⋯O hydrogen bonds and π–π inter­actions between pyridinium and benzene rings, with centroid–centroid distances in the range 3.516 (9)–3.553 (8) Å. The crystal is a twin with twin law, TWIN 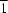 0 0 0 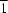 0 1 0 1. The monocation and dication are disordered with fractional site occupancy ratio of 0.75:0.25.

## Related literature

For bond-length data, see: Allen *et al.* (1987 ▶). For background to non-linear optical materials, see: Williams (1984 ▶). For related structures, see: Chantrapromma *et al.* (2009 ▶); Fun *et al.* (2009 ▶). For the stability of the temperature controller used in the data collection, see: Cosier & Glazer, (1986 ▶).
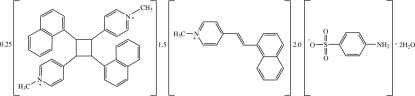


## Experimental

### 

#### Crystal data

1.5C_18_H_16_N^+^·0.25C_36_H_32_N_2_^2+^·2C_6_H_6_NO_3_S^−^·2H_2_O
*M* *_r_* = 873.04
Monoclinic, 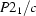

*a* = 6.6352 (4) Å
*b* = 14.8824 (8) Å
*c* = 20.9347 (13) Å
β = 97.921 (3)°
*V* = 2047.5 (2) Å^3^
*Z* = 2
Mo *K*α radiation
μ = 0.19 mm^−1^
*T* = 100 K
0.52 × 0.13 × 0.07 mm

#### Data collection

Bruker APEXII CCD area-detector diffractometer
Absorption correction: multi-scan (*SADABS*; Bruker, 2005 ▶) *T*_min_ = 0.906, *T*_max_ = 0.986
21302 measured reflections
4099 independent reflections
3550 reflections with *I* > 2σ(*I*)
*R*_int_ = 0.044

#### Refinement

*R*[*F* ^2^ > 2σ(*F* ^2^)] = 0.097
*wR*(*F* ^2^) = 0.275
*S* = 1.07
4099 reflections
440 parameters
300 restraints
H-atom parameters constrained
Δρ_max_ = 1.20 e Å^−3^
Δρ_min_ = −0.62 e Å^−3^

### 

Data collection: *APEX2* (Bruker, 2005 ▶); cell refinement: *SAINT* (Bruker, 2005 ▶); data reduction: *SAINT*; program(s) used to solve structure: *SHELXTL* (Sheldrick, 2008 ▶); program(s) used to refine structure: *SHELXTL*; molecular graphics: *SHELXTL*; software used to prepare material for publication: *SHELXTL* and *PLATON* (Spek, 2009 ▶).

## Supplementary Material

Crystal structure: contains datablocks global, I. DOI: 10.1107/S1600536809029730/ci2847sup1.cif

Structure factors: contains datablocks I. DOI: 10.1107/S1600536809029730/ci2847Isup2.hkl

Additional supplementary materials:  crystallographic information; 3D view; checkCIF report

## Figures and Tables

**Table 1:** Hydrogen-bond geometry (Å, °)

*D*—H⋯*A*	*D*—H	H⋯*A*	*D*⋯*A*	*D*—H⋯*A*
O1*W*—H1*W*1⋯O3	0.84	2.01	2.831 (7)	164
O1*W*—H2*W*1⋯O1^i^	0.84	2.04	2.853 (6)	164
N2—H2*B*⋯O1^ii^	0.86	2.23	3.018 (6)	153
N2—H2*C*⋯O2^iii^	0.86	2.23	2.991 (6)	147
C18—H18*A*⋯O1*W*	0.96	2.52	3.448 (13)	163
C18—H18*B*⋯O1*W*^iv^	0.96	2.21	3.168 (13)	173

Symmetry codes: (i) 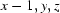; (ii) 
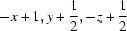
; (iii) 
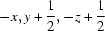
; (iv) 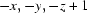.

**Fig. 1.**
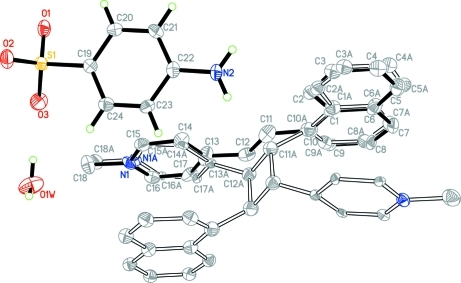
The molecular structure of the title compound, with 50% probability displacement ellipsoids and the atom-numbering scheme. The dication is shown in open bonds. Unlabelled atoms in the dication are related to the labelled atoms by the symmetry operation (- x, 1 -y, 1 - z). H atoms of cations were omitted for clarity. The monocation and dication have fractional occupancies of 0.75 and 0.25, respectively.

**Fig. 2.**
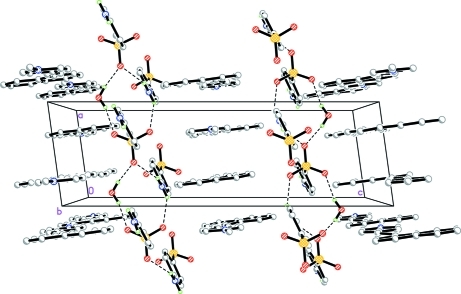
The crystal packing of the title compound viewed down the b axis. Hydrogen bonds are shown as dashed lines. The monocation with a fractional occupancy of 0.75 is shown while the dication with an occupancy of 0.25 has been omitted for clarity.

## supplementary crystallographic information

### Comment

The important requirement of NLO material is that the molecules have to align in noncentrosymmetric space group in the crystal (Williams, 1984). So the X-ray structure determination is a very important procedure to find out whether the compound is NLO active. During the course of our NLO research, we have previously reported crystal structures of (*E*)-1-methyl-4-[2-(2-naphthyl)vinyl]pyridinium iodide (I) (Fun *et al.*, 2009) and (*E*)-1-methyl-4-[2-(1-naphthyl)vinyl]pyridinium 4-bromobenzenesulfonate (II) (Chantrapromma *et al.*, 2009). These compounds were prepared by extending conjugated π-system which is a popular strategy to design NLO materials. In order to study the relation and effect of anions to the NLO properties, we attempted to synthesize (*E*)-1-methyl-4-[2-(2-naphthyl)vinyl]pyridinium 4-aminobenzenesulfonate (III) by replacing the iodide anion in (I) by 4-aminobenzenesulfonate. However, we got a co-crystal of the [2+2] cycloaddition product of (*E*)-1-methyl-4-[2-(2-naphthyl)vinyl]pyridinium with (III). The title compound crystallizes in the monoclinic centrosymmetric space group *P*2_1_/c, indicating that the second-order NLO properties are not present.

Fig. 1 shows the molecular structure of the title compound, which consists of one and a half C_18_H_16_N^+^ cation, one-quarter of the C_36_C_32_N_2_^2+^ dication, two C_6_H_6_NO_3_S^-^ anions and two water molecules. The monocation exists in the *E* configuration with respect to the C11═C12 double bond (C10—C11—C12—C13 = -179.9 (10)°) and is almost planar with dihedral angles between the pyridinium and C1–C6 and C1/C6–C10 rings being 3.1 (7) and 3.8 (8)°, respectively. The dication lies on an inversion center. The dihedral angle between the pyridinium and two aromatic C1A–C6A and C1A/C6A–C10A rings being 13 (2) and 14 (2)°, respectively, with the C10A–C11A–C12A–C13A torsion angle being -115 (3)°. Bond lengths are in normal ranges (Allen *et al.*, 1987) and comparable with those in related structures (Chantrapromma  *et al.*, 2009; Fun *et al*., 2009).

In the crystal structure, the anions and water molecules are linked into a chain along the *a* axis by O—H···O and N—H···O hydrogen bonds (Fig.2). The structure is further stabilized by C—H···O hydrogen bonds (Table 1) and π–π interactions between pyridinium and benzene rings [Cg1···Cg2^v^ = 3.553 (8) Å, Cg1···Cg2^vi^ = 3.530 (8) Å, Cg1···Cg3^v^ = 3.550 (9) Å and Cg1···Cg3^vi^ = 3.516 (9) Å; Cg1, Cg2 and Cg3 are centroids of the N1/C13-C17, C1-C6 and C1/C6-C10 rings, respectively. Symmetry code: (v) -x, 1-y, 1-z; (vi) 1-x, 1-y, 1-z].

### Experimental

Silver(I) 4-aminobenzenesulfonate (compound A) was prepared by mixing a solution of sulfanilic acid (0.12 g, 0.71 mmol) in hot methanol (50 ml) with a solution of sodium hydroxide (0.03 g, 0.71 mmol) in methanol (30 ml), followed by addition of a solution of silver nitrate (0.12 g, 0.71 mmol) in methanol (30 ml). A colourless solution together with black solid of AgI was obtained which was then filtered. The white solid of compound A was collected after allowing the filtrate to stand in air for a few days. (*E*)-1-Methyl-4-[2-(1-naphthyl)vinyl]pyridinium iodide (compound B) was prepared by the previous method (Chantrapromma *et al.*, 2009). The yellow solid of compound B (0.27 g, 0.71 mmol) was mixed with compound A (0.20 g, 0.71 mmol) in methanol (100 ml) and stirred for 30 minutes. The precipitate of silver iodide which formed was filtered and the filtrate was evaporated to give a yellow solid product. The yellow solid was repeatedly recrystallized for three times by dissolving the yellow solid in methanol and the solution was heated at 323 K to get a clear solution. The [2+2] cycloaddition of the (*E*)-1-methyl-4-[2-(1-naphthyl)vinyl]pyridinium occured upon heating. Green needle-shaped single crystals of the title compound suitable for *x*-ray structure determination were obtained from a methanol solution by slow evaporation at room temperature over a month (m.p. 513-514 K).

### Refinement

The fractional occupancies of the monocation and dication were initially refined to 0.746 (8) and 0.254 (2), respectively, and later for charge-balance they were fixed at 0.75 and 0.25. In the dication, the *U*_ij_ components of all atoms were restrained to approximate isotropic behaviour and atoms closer than 1.7 Å were restrained to have the same *U*_ij_ components. Same *U*_ij_ parameters were used for atoms C2 and C2A and also for C3 and C3A. In the dication, the naphthalene and pyridinium rings were restrained to be planar. All H atoms were positioned geometrically and allowed to ride on their parent atoms, with N-H = 0.86 Å and C-H = 0.93-0.98 Å. The *U*_iso_ values were constrained to be 1.5*U*_eq_ of the carrier atom for methyl H atoms and 1.2*U*_eq_(C) for the remaining H atoms. The highest residual electron density peak is located at 1.18 Å from C12 and the deepest hole is located at 0.74 Å from S1. The crystal is a twin with twin law, TWIN -1 0 0 0 -1 0 1 0 1 and BASF = 0.036 (1).

### Crystal data

**Table:**

1.5C_18_H_16_N^+^·0.25C_36_H_32_N_2_^2+^·2C_6_H_6_NO_3_S^−^·2H_2_O	*F*(000) = 920
*M**_r_* = 873.04	*D*_x_ = 1.416 Mg m^−^^3^
Monoclinic, *P*2_1_/*c*	Melting point = 513–514 K
Hall symbol: -P 2ybc	Mo *K*α radiation, λ = 0.71073 Å
*a* = 6.6352 (4) Å	Cell parameters from 4099 reflections
*b* = 14.8824 (8) Å	θ = 1.0–26.0°
*c* = 20.9347 (13) Å	µ = 0.19 mm^−^^1^
β = 97.921 (3)°	*T* = 100 K
*V* = 2047.5 (2) Å^3^	Needle, green
*Z* = 2	0.52 × 0.13 × 0.07 mm

### Data collection

**Table:**

Bruker APEXII CCD area-detector diffractometer	4099 independent reflections
Radiation source: sealed tube	3550 reflections with *I* > 2σ(*I*)
graphite	*R*_int_ = 0.044
φ and ω scans	θ_max_ = 26.0°, θ_min_ = 1.0°
Absorption correction: multi-scan (*SADABS*; Bruker, 2005)	*h* = −8→8
*T*_min_ = 0.906, *T*_max_ = 0.986	*k* = −16→18
21302 measured reflections	*l* = −25→23

### Refinement

**Table:**

Refinement on *F*^2^	Primary atom site location: structure-invariant direct methods
Least-squares matrix: full	Secondary atom site location: difference Fourier map
*R*[*F*^2^ > 2σ(*F*^2^)] = 0.097	Hydrogen site location: inferred from neighbouring sites
*wR*(*F*^2^) = 0.275	H-atom parameters constrained
*S* = 1.07	*w* = 1/[σ^2^(*F*_o_^2^) + (0.1017*P*)^2^ + 16.5792*P*] where *P* = (*F*_o_^2^ + 2*F*_c_^2^)/3
4099 reflections	(Δ/σ)_max_ = 0.001
440 parameters	Δρ_max_ = 1.20 e Å^−^^3^
300 restraints	Δρ_min_ = −0.61 e Å^−^^3^

### Special details

**Table:**

Experimental. The crystal was placed in the cold stream of an Oxford Cryosystems Cobra open-flow nitrogen cryostat (Cosier & Glazer, 1986) operating at 100.0 (1) K.
Geometry. All esds (except the esd in the dihedral angle between two l.s. planes) are estimated using the full covariance matrix. The cell esds are taken into account individually in the estimation of esds in distances, angles and torsion angles; correlations between esds in cell parameters are only used when they are defined by crystal symmetry. An approximate (isotropic) treatment of cell esds is used for estimating esds involving l.s. planes.
Refinement. Refinement of F^2^ against ALL reflections. The weighted R-factor wR and goodness of fit S are based on F^2^, conventional R-factors R are based on F, with F set to zero for negative F^2^. The threshold expression of F^2^ > 2sigma(F^2^) is used only for calculating R-factors(gt) etc. and is not relevant to the choice of reflections for refinement. R-factors based on F^2^ are statistically about twice as large as those based on F, and R- factors based on ALL data will be even larger.

### Fractional atomic coordinates and isotropic or equivalent isotropic displacement parameters (Å^2^)

**Table:**

	*x*	*y*	*z*	*U*_iso_*/*U*_eq_	Occ. (<1)
S1	0.3679 (2)	0.09002 (8)	0.28289 (6)	0.0218 (3)	
O1	0.5862 (6)	0.0903 (2)	0.28680 (19)	0.0275 (9)	
O2	0.2700 (6)	0.0497 (3)	0.2232 (2)	0.0298 (9)	
O3	0.2958 (7)	0.0516 (3)	0.3399 (2)	0.0352 (10)	
N2	0.1545 (7)	0.4776 (3)	0.2898 (2)	0.0245 (10)	
H2B	0.2400	0.5174	0.2807	0.029*	
H2C	0.0426	0.4940	0.3028	0.029*	
C19	0.2965 (8)	0.2043 (3)	0.2812 (2)	0.0197 (10)	
C20	0.4260 (8)	0.2701 (4)	0.2610 (2)	0.0227 (11)	
H20	0.5461	0.2529	0.2461	0.027*	
C21	0.3758 (8)	0.3600 (4)	0.2630 (3)	0.0226 (11)	
H21	0.4642	0.4028	0.2501	0.027*	
C22	0.1966 (8)	0.3877 (3)	0.2838 (2)	0.0197 (10)	
C23	0.0644 (8)	0.3219 (4)	0.3019 (2)	0.0212 (11)	
H23	−0.0585	0.3393	0.3148	0.025*	
C24	0.1135 (8)	0.2316 (4)	0.3009 (2)	0.0224 (11)	
H24	0.0242	0.1889	0.3134	0.027*	
N1	0.2543 (17)	0.1774 (6)	0.5190 (5)	0.024 (2)	0.75
C1	0.2403 (16)	0.6807 (6)	0.4633 (4)	0.0197 (19)	0.75
C2	0.2154 (18)	0.6500 (6)	0.3985 (4)	0.0223 (17)	0.75
H2	0.2124	0.5866	0.3900	0.027*	0.75
C3	0.1902 (17)	0.7092 (5)	0.3482 (5)	0.0228 (19)	0.75
H3	0.1750	0.6867	0.3048	0.027*	0.75
C4	0.191 (2)	0.8026 (7)	0.3597 (5)	0.025 (3)	0.75
H4	0.1721	0.8437	0.3240	0.030*	0.75
C5	0.215 (3)	0.8344 (7)	0.4214 (6)	0.028 (3)	0.75
H5	0.2177	0.8980	0.4290	0.033*	0.75
C6	0.240 (3)	0.7748 (6)	0.4755 (5)	0.022 (2)	0.75
C7	0.264 (4)	0.8077 (7)	0.5393 (6)	0.025 (3)	0.75
H7	0.2570	0.8712	0.5468	0.030*	0.75
C8	0.294 (3)	0.7491 (7)	0.5904 (5)	0.021 (2)	0.75
H8	0.3159	0.7714	0.6338	0.026*	0.75
C9	0.294 (2)	0.6548 (7)	0.5788 (4)	0.020 (2)	0.75
H9	0.3097	0.6144	0.6149	0.024*	0.75
C10	0.269 (2)	0.6203 (6)	0.5185 (4)	0.025 (2)	0.75
C11	0.2690 (12)	0.5210 (5)	0.5082 (4)	0.0316 (18)	0.75
H11	0.2690	0.5006	0.4647	0.038*	0.75
C12	0.2670 (11)	0.4559 (5)	0.5546 (4)	0.0280 (16)	0.75
H12	0.2651	0.4745	0.5984	0.034*	0.75
C13	0.2678 (15)	0.3597 (6)	0.5405 (4)	0.032 (2)	0.75
C14	0.2476 (19)	0.3249 (8)	0.4777 (5)	0.027 (3)	0.75
H14	0.2419	0.3647	0.4414	0.032*	0.75
C15	0.239 (3)	0.2339 (7)	0.4682 (6)	0.026 (3)	0.75
H15	0.2190	0.2103	0.4251	0.031*	0.75
C16	0.274 (2)	0.2081 (7)	0.5799 (5)	0.028 (3)	0.75
H16	0.2816	0.1666	0.6153	0.034*	0.75
C17	0.2813 (15)	0.2983 (7)	0.5916 (4)	0.025 (2)	0.75
H17	0.2945	0.3197	0.6352	0.030*	0.75
C18	0.259 (2)	0.0794 (7)	0.5068 (6)	0.035 (3)	0.75
H18A	0.1841	0.0665	0.4652	0.053*	0.75
H18B	0.1984	0.0482	0.5395	0.053*	0.75
H18C	0.3974	0.0602	0.5078	0.053*	0.75
N1A	0.285 (6)	0.1907 (16)	0.5264 (14)	0.018 (5)	0.25
C1A	0.239 (4)	0.6868 (16)	0.4553 (14)	0.024 (4)	0.25
C2A	0.208 (6)	0.6680 (19)	0.3881 (14)	0.0223 (17)	0.25
H2A	0.1985	0.6086	0.3742	0.027*	0.25
C3A	0.193 (5)	0.735 (2)	0.3437 (15)	0.0228 (19)	0.25
H3A	0.1732	0.7209	0.3000	0.027*	0.25
C4A	0.206 (7)	0.826 (2)	0.3629 (17)	0.024 (5)	0.25
H4A	0.1959	0.8710	0.3320	0.028*	0.25
C5A	0.233 (10)	0.8472 (19)	0.4267 (17)	0.023 (6)	0.25
H5A	0.2386	0.9073	0.4390	0.028*	0.25
C6A	0.254 (11)	0.7784 (17)	0.4752 (15)	0.025 (5)	0.25
C7A	0.285 (12)	0.801 (2)	0.5412 (16)	0.025 (6)	0.25
H7A	0.2940	0.8613	0.5538	0.030*	0.25
C8A	0.300 (10)	0.734 (2)	0.5867 (16)	0.026 (6)	0.25
H8A	0.3174	0.7491	0.6303	0.031*	0.25
C9A	0.290 (8)	0.643 (2)	0.5675 (15)	0.026 (6)	0.25
H9A	0.3136	0.5984	0.5990	0.031*	0.25
C10A	0.248 (7)	0.6176 (16)	0.5052 (15)	0.025 (5)	0.25
C11A	0.138 (4)	0.5304 (12)	0.4899 (10)	0.026 (4)	0.25
H11A	0.2028	0.5009	0.4561	0.031*	0.25
C12A	0.108 (3)	0.4637 (11)	0.5361 (9)	0.023 (3)	0.25
H12A	0.1363	0.4886	0.5797	0.028*	0.25
C13A	0.204 (4)	0.3737 (12)	0.5336 (10)	0.014 (4)	0.25
C14A	0.208 (5)	0.3306 (17)	0.4740 (11)	0.012 (5)	0.25
H14A	0.1831	0.3634	0.4359	0.015*	0.25
C15A	0.247 (8)	0.2405 (17)	0.4719 (14)	0.016 (6)	0.25
H15A	0.2481	0.2128	0.4322	0.019*	0.25
C16A	0.284 (6)	0.2299 (17)	0.5844 (13)	0.012 (5)	0.25
H16A	0.3106	0.1956	0.6218	0.014*	0.25
C17A	0.244 (4)	0.3190 (16)	0.5887 (11)	0.011 (5)	0.25
H17A	0.2423	0.3446	0.6292	0.014*	0.25
C18A	0.300 (6)	0.0916 (17)	0.5214 (19)	0.033 (8)	0.25
H18D	0.3278	0.0661	0.5639	0.049*	0.25
H18E	0.4090	0.0766	0.4974	0.049*	0.25
H18F	0.1747	0.0680	0.4998	0.049*	0.25
O1W	−0.0921 (7)	0.0188 (3)	0.3769 (2)	0.0447 (12)	
H1W1	0.0098	0.0320	0.3590	0.067*	
H2W1	−0.1996	0.0310	0.3525	0.067*	

### Atomic displacement parameters (Å^2^)

**Table:**

	*U*^11^	*U*^22^	*U*^33^	*U*^12^	*U*^13^	*U*^23^
S1	0.0227 (7)	0.0165 (6)	0.0267 (7)	0.0017 (5)	0.0054 (5)	0.0013 (5)
O1	0.034 (2)	0.0158 (18)	0.032 (2)	0.0041 (16)	0.0041 (17)	−0.0007 (16)
O2	0.031 (2)	0.022 (2)	0.035 (2)	−0.0017 (16)	0.0011 (17)	−0.0079 (17)
O3	0.049 (3)	0.025 (2)	0.034 (2)	0.0064 (18)	0.015 (2)	0.0102 (18)
N2	0.028 (2)	0.014 (2)	0.033 (2)	0.0018 (18)	0.008 (2)	−0.0024 (18)
C19	0.019 (2)	0.021 (3)	0.018 (2)	0.000 (2)	0.0002 (19)	0.001 (2)
C20	0.021 (3)	0.026 (3)	0.022 (3)	0.001 (2)	0.006 (2)	0.001 (2)
C21	0.022 (3)	0.021 (3)	0.025 (3)	−0.003 (2)	0.003 (2)	0.005 (2)
C22	0.030 (3)	0.018 (2)	0.010 (2)	0.001 (2)	−0.0016 (19)	0.0020 (18)
C23	0.021 (3)	0.024 (3)	0.020 (2)	0.003 (2)	0.007 (2)	0.000 (2)
C24	0.022 (3)	0.027 (3)	0.019 (2)	−0.001 (2)	0.002 (2)	0.000 (2)
N1	0.019 (5)	0.024 (4)	0.027 (4)	−0.009 (3)	0.001 (3)	−0.002 (3)
C1	0.014 (3)	0.025 (4)	0.020 (4)	−0.003 (3)	0.003 (3)	−0.007 (3)
C2	0.020 (3)	0.024 (4)	0.023 (4)	0.001 (3)	0.003 (3)	−0.002 (3)
C3	0.023 (3)	0.027 (5)	0.019 (3)	−0.003 (4)	0.002 (2)	−0.001 (4)
C4	0.021 (5)	0.032 (7)	0.021 (4)	0.000 (5)	−0.002 (3)	0.001 (4)
C5	0.029 (6)	0.025 (5)	0.028 (5)	0.001 (5)	0.000 (4)	−0.004 (4)
C6	0.014 (5)	0.027 (4)	0.027 (4)	0.002 (3)	0.002 (3)	−0.004 (3)
C7	0.029 (8)	0.016 (4)	0.029 (5)	0.001 (4)	0.004 (4)	−0.004 (3)
C8	0.020 (4)	0.016 (5)	0.026 (4)	0.000 (4)	−0.001 (3)	−0.010 (3)
C9	0.022 (4)	0.022 (5)	0.019 (4)	0.002 (3)	0.007 (4)	−0.007 (3)
C10	0.027 (5)	0.026 (4)	0.022 (6)	−0.003 (3)	0.006 (4)	−0.008 (3)
C11	0.029 (4)	0.040 (5)	0.024 (4)	0.008 (3)	−0.004 (3)	−0.004 (3)
C12	0.020 (4)	0.041 (4)	0.023 (4)	0.002 (3)	0.000 (3)	0.000 (3)
C13	0.034 (5)	0.031 (5)	0.027 (5)	0.015 (4)	−0.010 (4)	−0.001 (4)
C14	0.026 (7)	0.032 (5)	0.022 (4)	0.006 (4)	0.000 (4)	0.001 (4)
C15	0.020 (5)	0.033 (5)	0.023 (5)	−0.004 (4)	0.002 (4)	−0.005 (4)
C16	0.021 (5)	0.033 (6)	0.030 (5)	−0.004 (5)	0.005 (4)	0.002 (4)
C17	0.021 (5)	0.034 (7)	0.019 (4)	0.012 (4)	−0.001 (3)	0.001 (4)
C18	0.044 (7)	0.026 (5)	0.034 (6)	−0.007 (4)	−0.002 (5)	−0.002 (4)
N1A	0.018 (7)	0.017 (7)	0.020 (7)	−0.003 (6)	0.001 (5)	0.000 (5)
C1A	0.023 (6)	0.026 (6)	0.024 (6)	0.001 (5)	0.004 (5)	0.001 (5)
C2A	0.020 (3)	0.024 (4)	0.023 (4)	0.001 (3)	0.003 (3)	−0.002 (3)
C3A	0.023 (3)	0.027 (5)	0.019 (3)	−0.003 (4)	0.002 (2)	−0.001 (4)
C4A	0.022 (7)	0.026 (7)	0.022 (7)	−0.001 (6)	0.002 (5)	0.001 (6)
C5A	0.023 (7)	0.027 (8)	0.019 (8)	0.000 (6)	0.003 (6)	−0.001 (6)
C6A	0.024 (7)	0.026 (7)	0.024 (7)	0.001 (6)	0.004 (6)	0.000 (6)
C7A	0.026 (8)	0.025 (8)	0.025 (8)	0.000 (6)	0.004 (6)	0.000 (6)
C8A	0.024 (8)	0.026 (8)	0.028 (8)	−0.001 (6)	0.004 (6)	0.002 (6)
C9A	0.025 (8)	0.026 (8)	0.026 (8)	−0.002 (6)	0.004 (6)	0.002 (6)
C10A	0.025 (6)	0.029 (7)	0.023 (6)	−0.002 (5)	0.004 (5)	−0.003 (5)
C11A	0.026 (6)	0.027 (6)	0.025 (6)	−0.001 (5)	0.003 (5)	−0.001 (5)
C12A	0.025 (6)	0.025 (6)	0.021 (6)	0.002 (5)	0.005 (5)	0.001 (5)
C13A	0.016 (6)	0.013 (6)	0.014 (6)	0.002 (5)	0.005 (5)	0.006 (5)
C14A	0.012 (7)	0.012 (7)	0.013 (7)	−0.003 (5)	0.004 (5)	0.003 (6)
C15A	0.015 (8)	0.017 (8)	0.016 (8)	−0.003 (6)	0.001 (6)	0.002 (6)
C16A	0.010 (7)	0.014 (8)	0.012 (7)	0.002 (6)	0.003 (5)	0.004 (5)
C17A	0.012 (7)	0.010 (7)	0.012 (7)	0.004 (6)	0.001 (5)	0.004 (5)
C18A	0.031 (11)	0.033 (11)	0.034 (11)	−0.010 (8)	0.006 (8)	−0.007 (8)
O1W	0.041 (3)	0.061 (3)	0.034 (2)	0.017 (2)	0.012 (2)	0.015 (2)

### Geometric parameters (Å, °)

**Table:**

S1—O1	1.439 (4)	C16—C17	1.363 (12)
S1—O2	1.456 (4)	C16—H16	0.96
S1—O3	1.462 (4)	C17—H17	0.96
S1—C19	1.765 (5)	C18—H18A	0.96
N2—C22	1.376 (7)	C18—H18B	0.96
N2—H2B	0.86	C18—H18C	0.96
N2—H2C	0.86	N1A—C16A	1.348 (17)
C19—C24	1.395 (7)	N1A—C15A	1.354 (16)
C19—C20	1.406 (7)	N1A—C18A	1.484 (18)
C20—C21	1.381 (8)	C1A—C2A	1.421 (16)
C20—H20	0.93	C1A—C6A	1.425 (16)
C21—C22	1.385 (8)	C1A—C10A	1.463 (17)
C21—H21	0.93	C2A—C3A	1.358 (18)
C22—C23	1.401 (7)	C2A—H2A	0.93
C23—C24	1.384 (8)	C3A—C4A	1.407 (19)
C23—H23	0.93	C3A—H3A	0.93
C24—H24	0.93	C4A—C5A	1.361 (18)
N1—C16	1.344 (10)	C4A—H4A	0.93
N1—C15	1.350 (9)	C5A—C6A	1.435 (16)
N1—C18	1.481 (11)	C5A—H5A	0.93
C1—C2	1.419 (9)	C6A—C7A	1.411 (16)
C1—C6	1.423 (9)	C7A—C8A	1.373 (17)
C1—C10	1.457 (10)	C7A—H7A	0.93
C2—C3	1.364 (10)	C8A—C9A	1.422 (18)
C2—H2	0.96	C8A—H8A	0.93
C3—C4	1.411 (11)	C9A—C10A	1.348 (18)
C3—H3	0.96	C9A—H9A	0.93
C4—C5	1.364 (10)	C10A—C11A	1.500 (9)
C4—H4	0.96	C11A—C12A	1.421 (18)
C5—C6	1.431 (9)	C11A—C12A^i^	1.65 (3)
C5—H5	0.96	C11A—H11A	0.98
C6—C7	1.411 (9)	C12A—C13A	1.488 (18)
C7—C8	1.373 (10)	C12A—C11A^i^	1.65 (3)
C7—H7	0.96	C12A—H12A	0.98
C8—C9	1.424 (10)	C13A—C17A	1.406 (18)
C8—H8	0.96	C13A—C14A	1.407 (16)
C9—C10	1.351 (10)	C14A—C15A	1.368 (17)
C9—H9	0.96	C14A—H14A	0.93
C10—C11	1.493 (11)	C15A—H15A	0.93
C11—C12	1.374 (11)	C16A—C17A	1.359 (19)
C11—H11	0.96	C16A—H16A	0.93
C12—C13	1.462 (11)	C17A—H17A	0.93
C12—H12	0.96	C18A—H18D	0.96
C13—C17	1.401 (11)	C18A—H18E	0.96
C13—C14	1.402 (10)	C18A—H18F	0.96
C14—C15	1.368 (10)	O1W—H1W1	0.84
C14—H14	0.96	O1W—H2W1	0.84
C15—H15	0.96		
			
O1—S1—O2	112.1 (2)	C17—C16—H16	119.9
O1—S1—O3	113.2 (3)	C16—C17—C13	120.6 (8)
O2—S1—O3	112.5 (3)	C16—C17—H17	119.6
O1—S1—C19	105.2 (2)	C13—C17—H17	119.8
O2—S1—C19	107.1 (2)	N1—C18—H18A	109.5
O3—S1—C19	106.0 (2)	N1—C18—H18B	109.5
C22—N2—H2B	120.0	H18A—C18—H18B	109.5
C22—N2—H2C	120.0	N1—C18—H18C	109.5
H2B—N2—H2C	120.0	H18A—C18—H18C	109.5
C24—C19—C20	118.6 (5)	H18B—C18—H18C	109.5
C24—C19—S1	121.1 (4)	C16A—N1A—C15A	120.0 (18)
C20—C19—S1	120.2 (4)	C16A—N1A—C18A	120 (2)
C21—C20—C19	120.4 (5)	C15A—N1A—C18A	119.4 (19)
C21—C20—H20	119.8	C2A—C1A—C6A	118.2 (16)
C19—C20—H20	119.8	C2A—C1A—C10A	123.7 (18)
C20—C21—C22	121.3 (5)	C6A—C1A—C10A	118.0 (16)
C20—C21—H21	119.4	C3A—C2A—C1A	121.3 (19)
C22—C21—H21	119.4	C3A—C2A—H2A	119.4
N2—C22—C21	120.8 (5)	C1A—C2A—H2A	119.4
N2—C22—C23	120.8 (5)	C2A—C3A—C4A	120.9 (19)
C21—C22—C23	118.3 (5)	C2A—C3A—H3A	119.5
C24—C23—C22	121.2 (5)	C4A—C3A—H3A	119.5
C24—C23—H23	119.4	C5A—C4A—C3A	120 (2)
C22—C23—H23	119.4	C5A—C4A—H4A	120.0
C23—C24—C19	120.2 (5)	C3A—C4A—H4A	120.0
C23—C24—H24	119.9	C4A—C5A—C6A	121 (2)
C19—C24—H24	119.9	C4A—C5A—H5A	119.5
C16—N1—C15	121.5 (8)	C6A—C5A—H5A	119.5
C16—N1—C18	119.8 (8)	C7A—C6A—C1A	120.8 (17)
C15—N1—C18	118.6 (8)	C7A—C6A—C5A	120.5 (18)
C2—C1—C6	119.1 (7)	C1A—C6A—C5A	118.6 (16)
C2—C1—C10	123.1 (7)	C8A—C7A—C6A	119.4 (19)
C6—C1—C10	117.8 (7)	C8A—C7A—H7A	120.3
C3—C2—C1	121.0 (7)	C6A—C7A—H7A	120.3
C3—C2—H2	119.7	C7A—C8A—C9A	120 (2)
C1—C2—H2	119.3	C7A—C8A—H8A	119.9
C2—C3—C4	120.5 (7)	C9A—C8A—H8A	119.9
C2—C3—H3	119.4	C10A—C9A—C8A	122 (2)
C4—C3—H3	120.1	C10A—C9A—H9A	118.8
C5—C4—C3	120.1 (8)	C8A—C9A—H9A	118.8
C5—C4—H4	120.0	C9A—C10A—C1A	118.6 (17)
C3—C4—H4	119.9	C9A—C10A—C11A	118 (2)
C4—C5—C6	121.3 (8)	C1A—C10A—C11A	119.0 (18)
C4—C5—H5	119.8	C12A—C11A—C10A	124.7 (17)
C6—C5—H5	118.9	C12A—C11A—C12A^i^	91.9 (16)
C7—C6—C1	120.6 (7)	C10A—C11A—C12A^i^	117 (2)
C7—C6—C5	121.3 (8)	C12A—C11A—H11A	107.2
C1—C6—C5	118.1 (7)	C10A—C11A—H11A	107.2
C8—C7—C6	120.1 (7)	C12A^i^—C11A—H11A	107.2
C8—C7—H7	120.1	C11A—C12A—C13A	120.2 (16)
C6—C7—H7	119.7	C11A—C12A—C11A^i^	88.1 (16)
C7—C8—C9	119.8 (7)	C13A—C12A—C11A^i^	116.5 (17)
C7—C8—H8	120.3	C11A—C12A—H12A	110.1
C9—C8—H8	119.9	C13A—C12A—H12A	110.1
C10—C9—C8	122.0 (7)	C11A^i^—C12A—H12A	110.1
C10—C9—H9	118.9	C17A—C13A—C14A	116.1 (16)
C8—C9—H9	119.1	C17A—C13A—C12A	121.7 (16)
C9—C10—C1	119.6 (7)	C14A—C13A—C12A	120.1 (16)
C9—C10—C11	120.6 (7)	C15A—C14A—C13A	120.1 (18)
C1—C10—C11	119.9 (7)	C15A—C14A—H14A	120.0
C12—C11—C10	126.6 (7)	C13A—C14A—H14A	120.0
C12—C11—H11	116.6	N1A—C15A—C14A	121.6 (19)
C10—C11—H11	116.8	N1A—C15A—H15A	119.2
C11—C12—C13	123.2 (7)	C14A—C15A—H15A	119.2
C11—C12—H12	118.3	N1A—C16A—C17A	120.4 (19)
C13—C12—H12	118.5	N1A—C16A—H16A	119.8
C17—C13—C14	117.6 (8)	C17A—C16A—H16A	119.8
C17—C13—C12	119.1 (8)	C16A—C17A—C13A	121.9 (18)
C14—C13—C12	123.3 (8)	C16A—C17A—H17A	119.1
C15—C14—C13	119.9 (9)	C13A—C17A—H17A	119.1
C15—C14—H14	120.0	N1A—C18A—H18D	109.5
C13—C14—H14	120.0	N1A—C18A—H18E	109.5
N1—C15—C14	120.4 (9)	H18D—C18A—H18E	109.5
N1—C15—H15	119.8	N1A—C18A—H18F	109.5
C14—C15—H15	119.8	H18D—C18A—H18F	109.5
N1—C16—C17	120.1 (8)	H18E—C18A—H18F	109.5
N1—C16—H16	120.1	H1W1—O1W—H2W1	110.2
			
O1—S1—C19—C24	−156.2 (4)	C18—N1—C16—C17	176.2 (12)
O2—S1—C19—C24	84.3 (5)	N1—C16—C17—C13	0.3 (16)
O3—S1—C19—C24	−36.0 (5)	C14—C13—C17—C16	−0.3 (15)
O1—S1—C19—C20	22.6 (5)	C12—C13—C17—C16	177.2 (11)
O2—S1—C19—C20	−96.9 (4)	C6A—C1A—C2A—C3A	0(3)
O3—S1—C19—C20	142.9 (4)	C10A—C1A—C2A—C3A	177 (2)
C24—C19—C20—C21	2.4 (8)	C1A—C2A—C3A—C4A	0(2)
S1—C19—C20—C21	−176.4 (4)	C2A—C3A—C4A—C5A	0(5)
C19—C20—C21—C22	−1.1 (8)	C3A—C4A—C5A—C6A	2(7)
C20—C21—C22—N2	175.5 (5)	C2A—C1A—C6A—C7A	180 (4)
C20—C21—C22—C23	−1.0 (8)	C10A—C1A—C6A—C7A	2(6)
N2—C22—C23—C24	−174.7 (5)	C2A—C1A—C6A—C5A	1(6)
C21—C22—C23—C24	1.8 (7)	C10A—C1A—C6A—C5A	−176 (4)
C22—C23—C24—C19	−0.5 (8)	C4A—C5A—C6A—C7A	179 (5)
C20—C19—C24—C23	−1.7 (7)	C4A—C5A—C6A—C1A	−2(7)
S1—C19—C24—C23	177.2 (4)	C1A—C6A—C7A—C8A	0(8)
C6—C1—C2—C3	−0.4 (13)	C5A—C6A—C7A—C8A	179 (5)
C10—C1—C2—C3	−179.7 (9)	C6A—C7A—C8A—C9A	1(8)
C1—C2—C3—C4	0.9 (12)	C7A—C8A—C9A—C10A	−6(7)
C2—C3—C4—C5	−0.9 (17)	C8A—C9A—C10A—C1A	8(6)
C3—C4—C5—C6	0(2)	C8A—C9A—C10A—C11A	−149 (4)
C2—C1—C6—C7	179.9 (13)	C2A—C1A—C10A—C9A	176 (3)
C10—C1—C6—C7	−1(2)	C6A—C1A—C10A—C9A	−6(5)
C2—C1—C6—C5	−0.1 (19)	C2A—C1A—C10A—C11A	−27 (4)
C10—C1—C6—C5	179.3 (13)	C6A—C1A—C10A—C11A	151 (4)
C4—C5—C6—C7	−179.9 (16)	C9A—C10A—C11A—C12A	−12 (5)
C4—C5—C6—C1	0(2)	C1A—C10A—C11A—C12A	−170 (2)
C1—C6—C7—C8	2(2)	C9A—C10A—C11A—C12A^i^	101 (4)
C5—C6—C7—C8	−178.0 (16)	C1A—C10A—C11A—C12A^i^	−56 (4)
C6—C7—C8—C9	−2(2)	C10A—C11A—C12A—C13A	−115 (3)
C7—C8—C9—C10	1(2)	C12A^i^—C11A—C12A—C13A	120 (2)
C8—C9—C10—C1	−0.1 (19)	C10A—C11A—C12A—C11A^i^	125 (3)
C8—C9—C10—C11	−179.9 (13)	C12A^i^—C11A—C12A—C11A^i^	0.000 (2)
C2—C1—C10—C9	179.2 (10)	C11A—C12A—C13A—C17A	156 (2)
C6—C1—C10—C9	−0.2 (17)	C11A^i^—C12A—C13A—C17A	−100 (2)
C2—C1—C10—C11	−1.0 (15)	C11A—C12A—C13A—C14A	−41 (3)
C6—C1—C10—C11	179.7 (13)	C11A^i^—C12A—C13A—C14A	63 (2)
C9—C10—C11—C12	9.0 (18)	C17A—C13A—C14A—C15A	0.4 (19)
C1—C10—C11—C12	−170.9 (9)	C12A—C13A—C14A—C15A	−163 (3)
C10—C11—C12—C13	−179.9 (10)	C16A—N1A—C15A—C14A	0(3)
C11—C12—C13—C17	175.2 (8)	C18A—N1A—C15A—C14A	172 (4)
C11—C12—C13—C14	−7.4 (14)	C13A—C14A—C15A—N1A	−1(3)
C17—C13—C14—C15	0.9 (15)	C15A—N1A—C16A—C17A	0(3)
C12—C13—C14—C15	−176.6 (12)	C18A—N1A—C16A—C17A	−172 (4)
C16—N1—C15—C14	1.4 (18)	N1A—C16A—C17A—C13A	−1(4)
C18—N1—C15—C14	−175.7 (13)	C14A—C13A—C17A—C16A	0(3)
C13—C14—C15—N1	−1.4 (18)	C12A—C13A—C17A—C16A	164 (3)
C15—N1—C16—C17	−0.9 (16)		

Symmetry codes: (i) −*x*, −*y*+1, −*z*+1.

### Hydrogen-bond geometry (Å, °)

**Table:**

*D*—H···*A*	*D*—H	H···*A*	*D*···*A*	*D*—H···*A*
O1W—H1W1···O3	0.84	2.01	2.831 (7)	164
O1W—H2W1···O1^ii^	0.84	2.04	2.853 (6)	164
N2—H2B···O1^iii^	0.86	2.23	3.018 (6)	153
N2—H2C···O2^iv^	0.86	2.23	2.991 (6)	147
C18—H18A···O1W	0.96	2.52	3.448 (13)	163
C18—H18B···O1W^v^	0.96	2.21	3.168 (13)	173

Symmetry codes: (ii) *x*−1, *y*, *z*; (iii) −*x*+1, *y*+1/2, −*z*+1/2; (iv) −*x*, *y*+1/2, −*z*+1/2; (v) −*x*, −*y*, −*z*+1.
